# Posterior Reversible Encephalopathy Syndrome with Bilateral Independent Epileptic Foci Precipitated By Guillain-Barrè Syndrome

**DOI:** 10.1155/2016/5913840

**Published:** 2016-06-15

**Authors:** Rosario Rossi, Maria Valeria Saddi, Alessandro Mela, Anna Ticca

**Affiliations:** Unit of Neurology, San Francesco Hospital, Via Mannironi, 08100 Nuoro, Italy

## Abstract

We report the case of a 56-year-old woman who developed status epilepticus (SE) related to independent occipital foci as clinical manifestation of posterior reversible encephalopathy syndrome (PRES) in the background of Guillain-Barrè syndrome (GBS). SE resulted from a series of focal seizures clinically characterized by left- and rightward deviations of the head and consequent oculoclonic movements. Electroencephalography recorded independent seizure activity in both occipital regions with alternate involvement of the two cerebral hemispheres. The epileptic foci corresponded topographically to parenchymal abnormalities of PRES in the occipital lobes. The manifestation of bilateral, independent occipital seizures with alternate deviations of the head and oculoclonic movements, previously not reported in patients with PRES, highlights the acute epileptogenicity of the cerebral lesions in this syndrome. Despite the variable clinical expression of seizures due to occipital damage in PRES, the development of independent seizure activity in both occipital lobes might represent a distinctive epileptic phenomenon of this encephalopathy.

## 1. Introduction

Epileptic seizures are a frequent clinical manifestation of posterior reversible encephalopathy syndrome (PRES). This clinicoradiological entity, initially described by Hinchey et al. with the term reversible posterior leukoencephalopathy syndrome [1], may be associated with a variety of predisposing conditions, including Guillain-Barrè syndrome (GBS) [2, 3]. Seizures classically arise in a clinical context of headache, vomiting, visual loss, and mental abnormalities but may also represent the only symptom of the encephalopathy [4]. A few reports suggest that occipital lobe seizures may typically occur in PRES because of the predominant posterior distribution of the parenchymal damage [4, 5]. Nonetheless, there is still no evidence of epileptic disorders with specific characteristics in this syndrome.

## 2. Case Presentation

**Figure 1:**
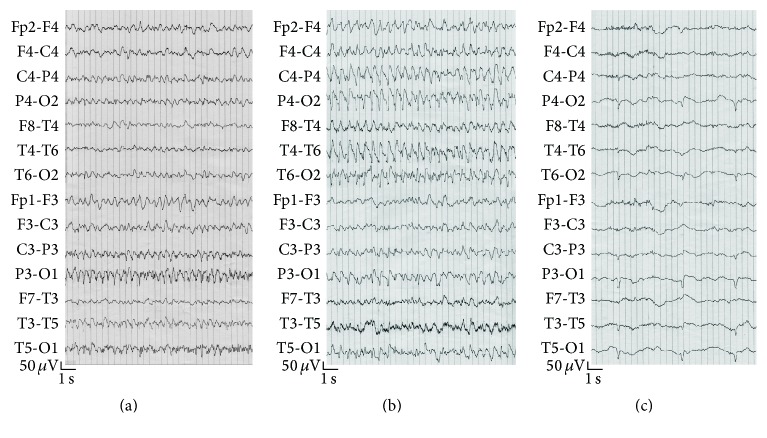
Independent seizure activity in the posterior cerebral regions. (a) Epileptic abnormalities in the left occipital area. (b) Rhythmic sharp waves in the right temporoparietooccipital regions. Leftward deviation of the head and oculoclonic movements were noted during the discharge. (c) Persistence of periodic sharp waves in both occipital regions after cessation of SE.

**Figure 2:**
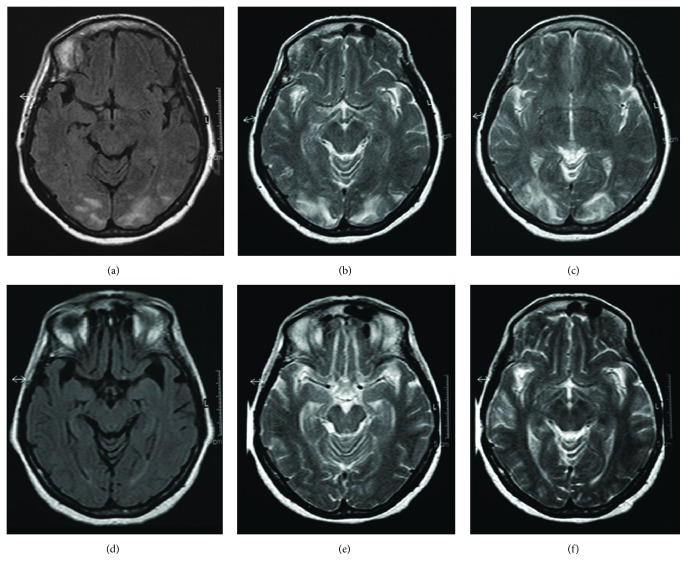
(a, b, c) MRI at the clinical onset of PRES. FLAIR (a) and T2-weighted (b, c) images indicate symmetric hyperintense abnormalities in the occipital lobes. (d, e, f) MRI four weeks after the clinical onset of PRES. FLAIR (d) and T2-weighted (e, f) images show regression of the occipital abnormalities.

A 56-year-old woman presented at our hospital with acute lumbar pain that radiated to the lower limbs and new-onset arterial hypertension. The symptoms had started 3 days before the admission. On the second day in the hospital, as blood pressure levels reached 200/110 mmHg, the patient also developed sinus tachyarrhythmia, paresthesia in the lower extremities, headache, vomiting, blurring of vision, and focal epileptic seizures that resulted in status epilepticus (SE). The seizure semiology was characterized by an impairment of consciousness and lateral deviations of the head and eyes with consequent oculoclonic movements that in one case led to a secondary generalization. Particularly, the occurrence of different seizures with left- and rightward deviations of the head and eyes suggested the presence of bilateral, independent epileptic activity. Electroencephalography (EEG) recorded a series of focal epileptic discharges that alternated over the two posterior cerebral regions in close but separated periods of time (Figures 1(a) and 1(b)). The discharges developed in each occipital area in the form of fast activity and consequent high-amplitude (up to 160 *μ*V), rhythmic sharp waves that frequently spread to the homolateral temporoparietal and central regions (Figure 2(b)). Occipital seizures originating in the two cerebral hemispheres occasionally overlapped in time. Interictal EEG displayed slowing of the background activity and periodic sharp waves in the occipital regions. SE was interrupted with intravenous administration of diazepam. Periodic sharp waves persisted in both occipital regions after final cessation of seizures (Figure 1(c)) and disappeared 2 days later. After recovering from SE, the patient continued to suffer from visual loss, vomiting, and lumbar pain. A computed tomography scan and magnetic resonance imaging (MRI) of the brain showed abnormalities typical of PRES in the occipital lobes (Figures 2(a), 2(b), and 2(c)). On the fifth day in the hospital, the patient developed flaccid tetraparesis and became unable to walk. After this event, a diagnosis of GBS was achieved with the support of electroneurography and investigations of the cerebrospinal fluid, which, respectively, revealed the disappearance of F waves and an albuminocytological dissociation. Arterial pressure progressively decreased and normalized with appropriate therapy in 9 days. A second MRI of the brain performed 4 weeks after the first examination showed a complete regression of the occipital abnormalities (Figures 2(d), 2(e), and 2(f)). The patient was finally discharged from the hospital with no medication for hypertension or epilepsy. Her blood pressure remained in the normal range and no seizures were reported during the following year.

## 3. Discussion

The patient presented with a confusing combination of peripheral and central neurologic symptoms due to the coexistence of GBS and PRES, an unusual clinical association that remarks the potential pathogenic relationship between the two syndromes. Considering the clinical context and timing of the symptoms, it is assumable that arterial hypertension developed acutely during a phase of GBS-related autonomic dysfunction and subsequently precipitated PRES. The normalization of arterial pressure despite the absence of treatment during the follow-up period supports the assumption of an acute hypertension as the precipitating factor for PRES.

Apart from the classic presentation of headache, vomiting, and visual loss, an unusual association of versive seizures characterized the clinical manifestation of PRES. Although epileptic disorders are frequently reported in this syndrome [1, 6], it is remarkable that SE in our patient resulted from a series of focal seizures with independent origins in the posterior region of each cerebral hemisphere. The observation of different seizures with left- and rightward deviations of the head and eyes is consistent with the EEG recordings of epileptic discharges in each posterior cerebral region at separate periods and demonstrates the activity of bilaterally symmetric, but independent, epileptic foci. Furthermore, the neuroradiological finding of symmetric occipital lesions indicates a clear pathogenic relationship between the seizure activity and parenchymal abnormalities of PRES. The EEG pattern of SE related to independent occipital foci corresponds to that shown in a previous case report of PRES not associated with GBS [7]. In this case, SE developed subclinically in a patient with metabolic abnormalities and prolonged disturbance of consciousness after a sequence of generalized tonic-clonic seizures. To the best of our knowledge, similar EEG characteristics of SE have been described in only two other reports, respectively, concerning two adult patients with hypertensive encephalopathy [8] and two pediatric patients with SE amauroticus related to PRES [9]. These case studies describe different correlates of ictal EEG abnormalities in the posterior cerebral regions, highlighting the variable clinical expression of seizures related to the bilateral occipital damage in PRES. Regarding hypertensive encephalopathy, Aguglia et al. reported subtle SE with bilateral posterior distribution in a comatose patient with concomitant renal failure and provided EEG recordings of independent occipital discharges with secondary generalization in another patient with focal motor seizures and consequent bilateral convulsions [8]. In the pediatric cases described by Muro et al., amaurosis was the main clinical manifestation of bilateral, independent occipital SE [9]. A major number of reports have documented the occurrence of occipital lobe seizures [4, 5, 10–12] and SE related to occipital localizations in PRES [13, 14] without demonstrating independent seizure activity on both sides of the brain. Considering the frequent symmetric distribution of the occipital damage in PRES, which is uncommon to see in other brain disorders, we believe that the development of acute independent epileptic foci in both occipital lobes represents a characteristic epileptic phenomenon of this syndrome. The association of bilateral, independent occipital seizures with alternate deviations of the head and eyes, previously not reported in patients with PRES, highlights the acute epileptogenicity of the parenchymal lesions, indicating the potential coexistence of different seizure disorders in this syndrome. The EEG finding of periodic sharp waves denotes a subclinical epileptic abnormality associated with the concluding phase of SE and confirms the occipital localizations of the epileptic foci (Figure 1(c)). Despite the bilateral synchronous presentation in our patient, this finding could be referred to as paroxysmal lateralized epileptiform discharges (PLEDs), an EEG pattern usually associated with focal brain damage that could mark the interictal period of seizures related to PRES, as also previously reported [5–7, 15].

## 4. Conclusion

SE resulting from bilateral, independent occipital seizures with alternate deviations of the head and oculoclonic movements may characterize the symptomatology of PRES. Despite the variable clinical expression of seizures related to occipital damage in PRES, the development of independent seizure activity in both occipital lobes might represent a distinctive epileptic phenomenon of this syndrome.
